# GIS-based hydrodynamic modeling for urban flood mitigation in fast-growing regions: a case study of Erbil, Kurdistan Region of Iraq

**DOI:** 10.1038/s41598-023-36138-9

**Published:** 2023-06-01

**Authors:** Andam Mustafa, Michał Szydłowski, Mozafar Veysipanah, Hasan Mohammed Hameed

**Affiliations:** 1 Faculty of Civil and Environmental Engineering, Gdańsk University of Technology, Narutowicza 11/12, 80-233 Gdańsk, Poland; 2 Department of Physical Geography and Ecosystem Science, Lund University, Lund, Sweden; 3 College of Engineering, Geomatics (Surveying) Engineering, Salahaddin University-Erbil, Erbil, 44001 Iraq

**Keywords:** Climate-change mitigation, Environmental impact, Hydrology, Natural hazards, Civil engineering

## Abstract

Floods threaten urban infrastructure, especially in residential neighborhoods and fast-growing regions. Flood hydrodynamic modeling helps identify flood-prone locations and improve mitigation plans' resilience. Urban floods pose special issues due to changing land cover and a lack of raw data. Using a GIS-based modeling interface, input files for the hydrodynamic model were developed. The physical basin's properties were identified using soil map data, Land Use Land Cover (LULC) maps, and a Digital Elevation Model (DEM). So, the HEC-RAS 2-D hydrodynamic model was developed to estimate flood susceptibility and vulnerability in Erbil, Iraq. The case study examines the quality of flood modeling results using different DEM precisions. Faced with the difficulty, this study examines two building representation techniques: Building Block (BB) and Building Resistance (BR). The work presented here reveals that it is possible to apply the BR technique within the HEC-RAS 2-D to create urban flood models for regions that have a lack of data or poor data quality. Indeed, the findings confirmed that the inundated areas or areas where water accumulated in past rainfall events in Erbil are the same as those identified in the numerical simulations. The study's results indicate that the Erbil city is susceptible to flood hazards, especially in areas with low-lying topography and substantial precipitation. The study's conclusions can be utilized to plan and develop flood control structures, since it identified flood-prone areas of the city.

## Introduction

Floods have developed into an event that people in developing countries regard as routine during the rainy season. Flash floods caused by climate change are one of the most common causes of fatalities and property damage in the twenty-first century. Recently, frequent urban flash floods have become the most popular topic among scientists, engineers, and local authorities in prone regions. Droughts and floods have historically been the most deadly natural disasters^1^. Aptly, the effects of flooding are expected to worsen as the world's population grows, the economy grows, and climate change^2^. Samanta, et al.^3^ classified floods into three types: flash floods that last a few hours; floods that last several hours to several days; and floods that occur gradually over a relatively long period of time. Cities in general have become locations that are more prone to be flooded as a result of continued urbanization and population growth^4^. Therefore, even though these natural disasters were unavoidable, it is necessary to investigate flood hazards in order to better manage them going forward. Flood hazard assessment and modeling, in conjunction with inundation maps, can be of assistance to climate experts and scientists in this regard.

Building detailed flood inundation maps is typically done through the use of hydrodynamic models. A significant amount of data, time, and computational resources are required for the development of a hydrodynamic model for a large hydrological basin, all of which must be available at the same time^5^. In developing countries, obtaining archived data for rainfall-runoff modelling, high-resolution DEM for topography and accurate LULC for basin characteristics is a challenge. As Loudyi and Kantoush^6^ pointed out, a serious challenge for flood risk assessment in the Middle East and North Africa (MENA) region is the unavailability and sometimes unreliability of data, particularly for the calibration and validation of flood models. There are some approaches that could be considered to fill such gaps, for example, as part of hydrological and hydrodynamic modeling of urban floods in Erbil, previously LULC from Landsat satellite images were prepared for the study area^7^. Furthermore, the availability of such data allows a more precise description of hydrological processes occurring in the real world, paving the way for a more numerical approximation of the processes involved in urban flooding. As a result of historical efforts to mitigate flood impacts, flood loss and damage in developed countries, particularly when measured in terms of the number of fatalities, is generally less severe than in developing countries^8^.

Researchers in many parts of the world have attempted to use geospatial techniques to model floods in urban and rural areas^9–16^ for surface and ground water detection^17,18^, analyzing rainfall^19–21^, LULC analysis^22–27^, and finally flood risk mapping and analysis^28–32^. A model that utilizes artificial neural networks (ANN), geographic information systems (GIS), and remote sensing (RS) was developed by^33,34^ to estimate ground water recharge mapping in the Iraqi Western Desert. Using a long-term perspective, a group of Polish researchers presented a method for analyzing the impact of urbanization on the frequency of flooding inside an urban catchment^35^. The integration of GIS remote sensing data and survey data with the ANN was used by^36^ to generate a hydrological soil group map for Alghadaf Wadi in Iraq, providing timely, fast, and relatively cheap data for mapping and monitoring soil texture. Samela, et al.^14^ developed a GIS tool that is able to delineate areas that are prone to flooding in an efficient and cost-effective manner. El-Saoud and Othman^37^ estimated flash floods in Makkah's catchment region utilizing spatial parameter distribution and mathematical hydrological and hydrodynamic modelling. This study created a flash flood risk map. SZYDLOWSKI^38^ presented flash flood propagation computations in natural and urban areas, shallow water equations are used to model free surface unsteady water flow. There are a variety of 2-D numerical models and software packages available, each with its own set of capabilities and developed by a variety of different developers; some must be purchased, while others are free and open-source^39^. Glenis, et al.^40^ presented and validated City Catchment Analysis Tool – CityCAT, a novel software for modeling, analysis, and visualization of surface water flooding. It consists of a 2-D overland flow routing model that provides quick evaluation of combined pluvial and fluvial urban flood risk and the effects of various flood mitigation strategies. Macalalad, et al.^41^ set up the Liuxihe model in order to develop a forecasting scheme and simulate the observed flood process. The study reproduced past flash flooding events and demonstrated a significant correlation between past dense storm events and their respective simulated river discharges. With the help of a 2-D rainfall-runoff simulation at the basin scale, Costabile, et al.^42^ investigated the performance and capabilities of the HEC-RAS 2-D model. Mustafa and Szydłowski^43^ demonstrated how different building representation techniques and hydrodynamic models can influence the results of urban flood simulations using the HEC-RAS 2-D package. Both BB and BR can be applied in HEC-RAS 2-D. Alipour, et al.^44^ examined HEC-RAS 2-D's configuration factors and parameters. They investigated the effects of several model configuration factors, such as the floodplain and channel roughness coefficients, terrain and mesh size, and river boundary conditions, on the dynamics of water levels, maximum water level and flood extent.

Following the invasion of Iraq by the coalition forces in April 2003, Erbil, the capital of the Kurdistan Region of Iraq (KRI), became the focal point of development in the region and is now regarded as an important political, economic, and administrative center not only for Iraq but also for the neighboring countries^45^. KRI has taken steps to develop its oil and gas industry and attract investment from international oil companies since 2009, in order to increase its capacity and prepare for a gradual transition to independence^46^. Rapid urbanization is one of the main causes of flash floods in Erbil. As a result, the patterns of LULC in Erbil have changed significantly over the last two decades, particularly in the urban areas^7^. The most important factor that has a significant impact on creating the danger of flash flooding is the improper use of land for urbanization where the paths of the waterways have been closed. In the aftermath of this, the number of urban flash floods has also increased significantly^47^. Changes and developments to the land surface within cities will have an effect not only on the likelihood of increased flooding, but also on urban water management generally^48^. On the other hand, there have been studies that have linked the increasing frequency of floods to the impact of climate change^49,50^. For applications in urban settings, high temporal resolution of precipitation, such as sub-daily, is required for establishing a relationship between extreme precipitation Intensity–Duration–Frequency IDF^51–53^. When it comes to developing countries such as Iraq, the number and distribution of stations capable of recording rainfall on a shorter time scale are limited. The Indian Meteorological Department (IMD) equation was used to determine the rainfall data for periods shorter than 24 h, and IDF curves and empirical IDF formulas for the city of Erbil were developed as a result of these limitations^54^. To assess flood risk, scientists consider the likelihood and severity of a flood in a specific area at a specific time, along with the potential consequences^55–57^.

From 1950 to 2010, the number of people who died in Iraq as a result of flooding was only 11^1^. In the fourth quarter of 2021, Erbil was struck by flash floods on October 30 and December 17, the latter of which was particularly devastating, resulting in the deaths of 12 people and the disappearance of the body of a 10-month-old child for almost two months. Another flash flood hit Erbil on January 13, 2022, approximately one month after the previous one. Specifically, this one was located mainly within the study area that we modeled in HEC-RAS 2-D in this paper. Flash floods are more common in areas with a dry climate and impervious terrain because lack of pervious surface or vegetation allows torrential rains to flow overland rather than infiltrate into the ground. According to the available evidence, the city has experienced three floods in the span of just three months. According to a press release from the province of Erbil, more than 7000 people were declared to be in a state of special calamity^58^, and the total flood damage in Erbil was estimated to be $14,5 million^59^. The economic impact of recent flash floods has increased dramatically. Despite the devastation caused by Erbil's flash floods, the study area's summer fresh-water shortage makes flash floods a valuable source of water.

This paper addresses a critical issue of urban flash floods in Erbil and presents a new approach to model and identify flood-prone areas using HEC-RAS 2-D hydrodynamic model, where there has been no such work previously. The novelty of the work lies in discussing the challenges of accurately assessing and modeling flood hazards in this area, particularly regarding the availability and reliability of data. Previous studies on stormwater management in Erbil have been lacking, and there is a need to establish sustainable strategies to mitigate the adverse effects of flooding on the socio-economic fabric of society. The study highlights the importance of accurate topographic representation using a DEM of different precision to improve the flood modeling results. The impact of floods on society is also discussed, emphasizing the need for effective management strategies. This study is the first of its kind in the study area, and it is expected to provide valuable insights into developing sustainable urban stormwater management practices.

## Materials and methods

### Study area

**Figure 1:**
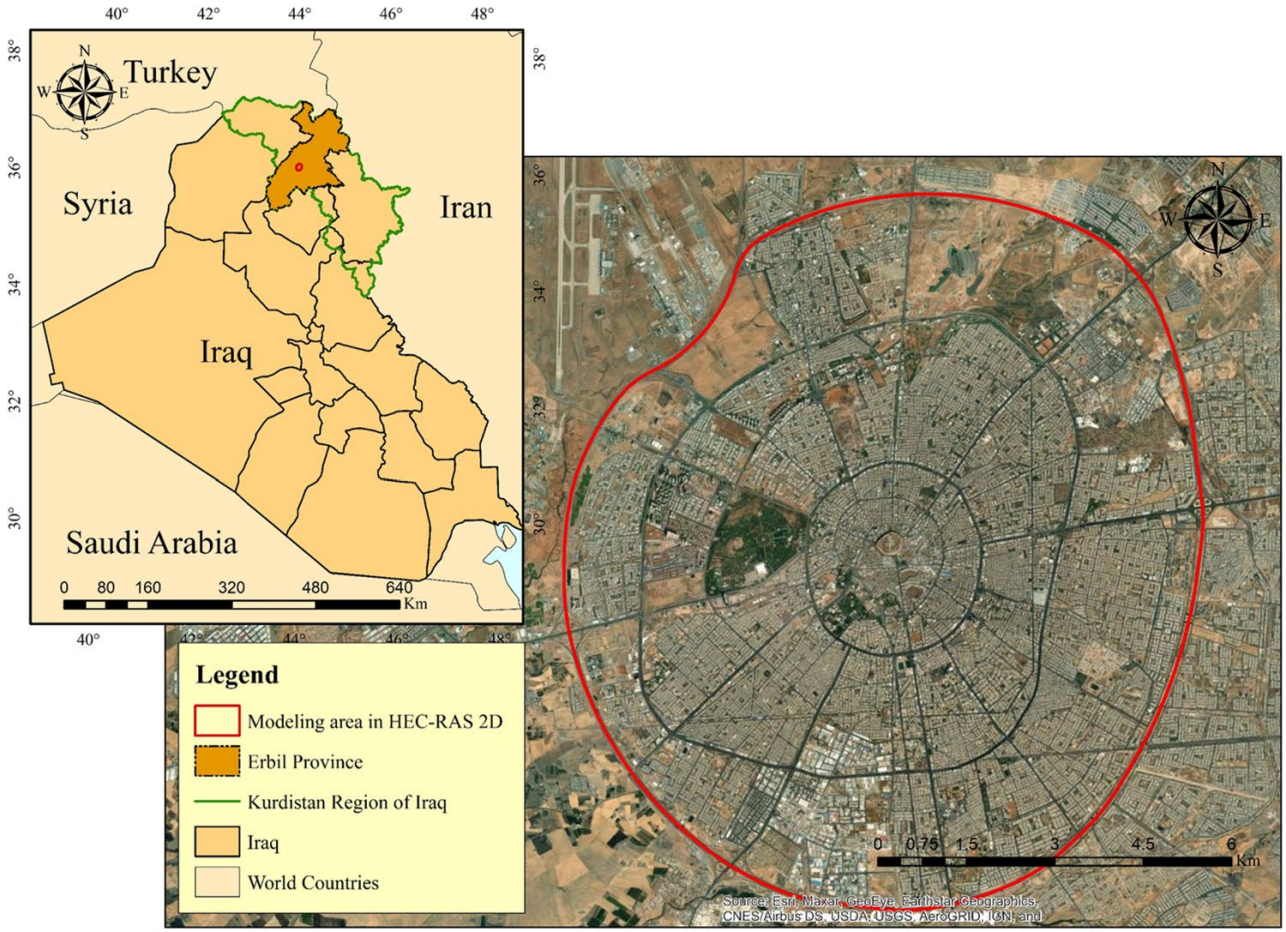
Study area. Base imagery sources: Esri, DigitalGlobe, GeoEye, i-cubed, USDA FSA, USGS, AEX, Getmapping, Aerogrid, IGN, IGP, swisstopo, and the GIS User Community. These maps were processed and generated in ArcGIS 10.7 software ( © ESRI, URL: https://www.esri.com/en-us/arcgis/products/index).

Erbil (also known as Hawler in the Kurdish language) is the capital city of the Kurdistan Region in northern Iraq, and it is the region's largest city by population. Erbil, Iraq's second-largest city in the north of the country after Mosul, is the region's economic hub. In 2020, it was expected to have a population of 2,254,422 inhabitants and more than one million of them living in the city center. Erbil Province has a total land area of 14,873.68 km^2^^2,60^. The investigation will be focused on the central district of Erbil. In the hydrodynamic modeling using HEC-RAS 2-D, the area that was considered was within the 120 m ring road (circular highway around the city), which is approximately 102.38 km^2^. Due to the fact that a DEM with an acceptable resolution is only available within this boundary, only this area is considered in the modeling as the study area in HEC-RAS 2-D (Fig. 1). In general, Erbil Province has a semi-arid continental climate, which is characterized by hot and dry summers and cold and wet winters, owing to its geographic location on the Arabian Plate. It is only possible to consider this climate for the city center because the mountainous region to the north and northeast of the province, which has a Mediterranean climate, cannot be considered. More detailed information can be found in^47,61,62^.

### Data availability and processing

**Figure 2:**
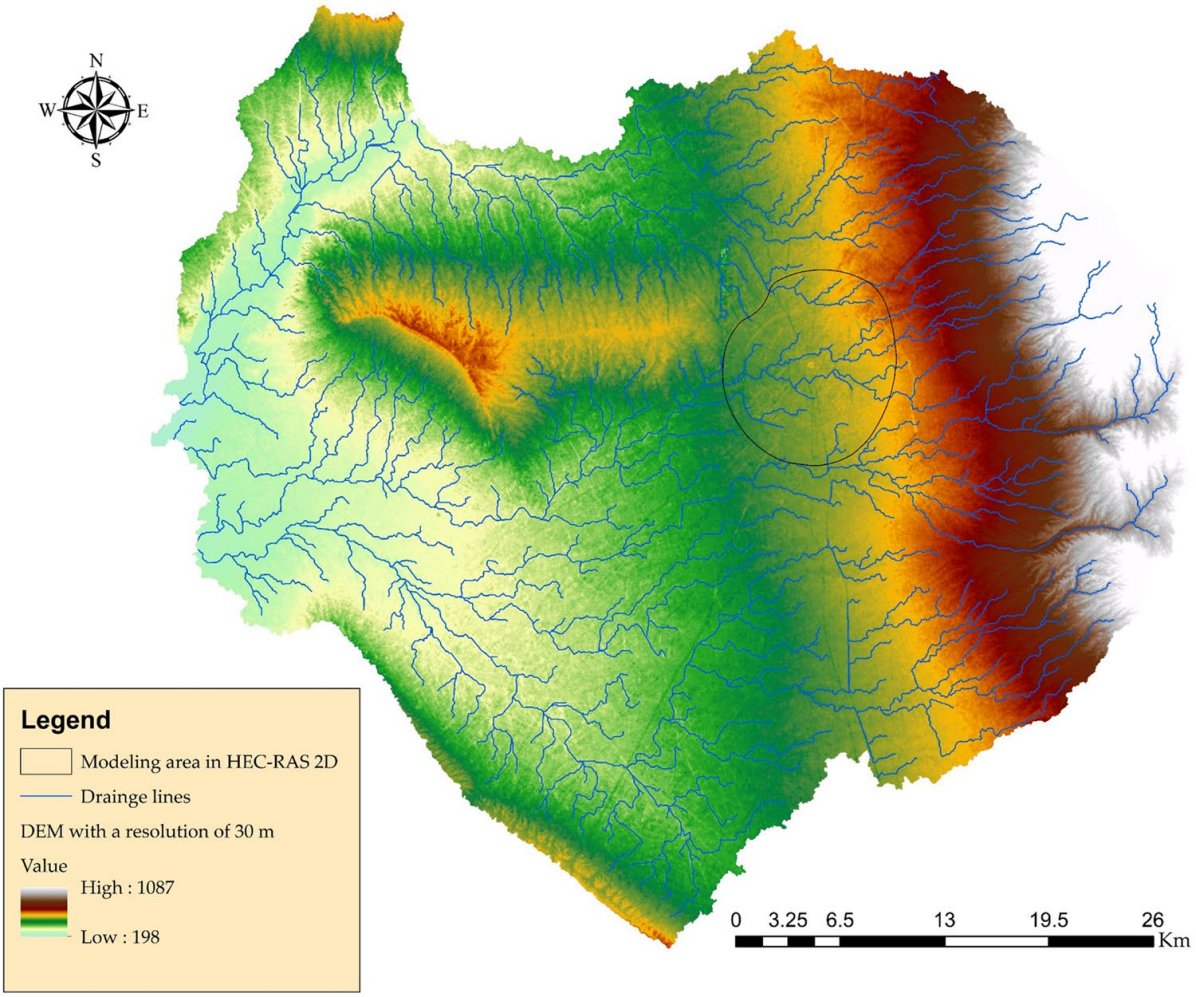
Delineation of watershed using a Digital Elevation Model (DEM) with a resolution of 30 m. This map is processed and generated in ArcGIS 10.7 software ( © ESRI, URL: https://www.esri.com/en-us/arcgis/products/index).

**Figure 3:**
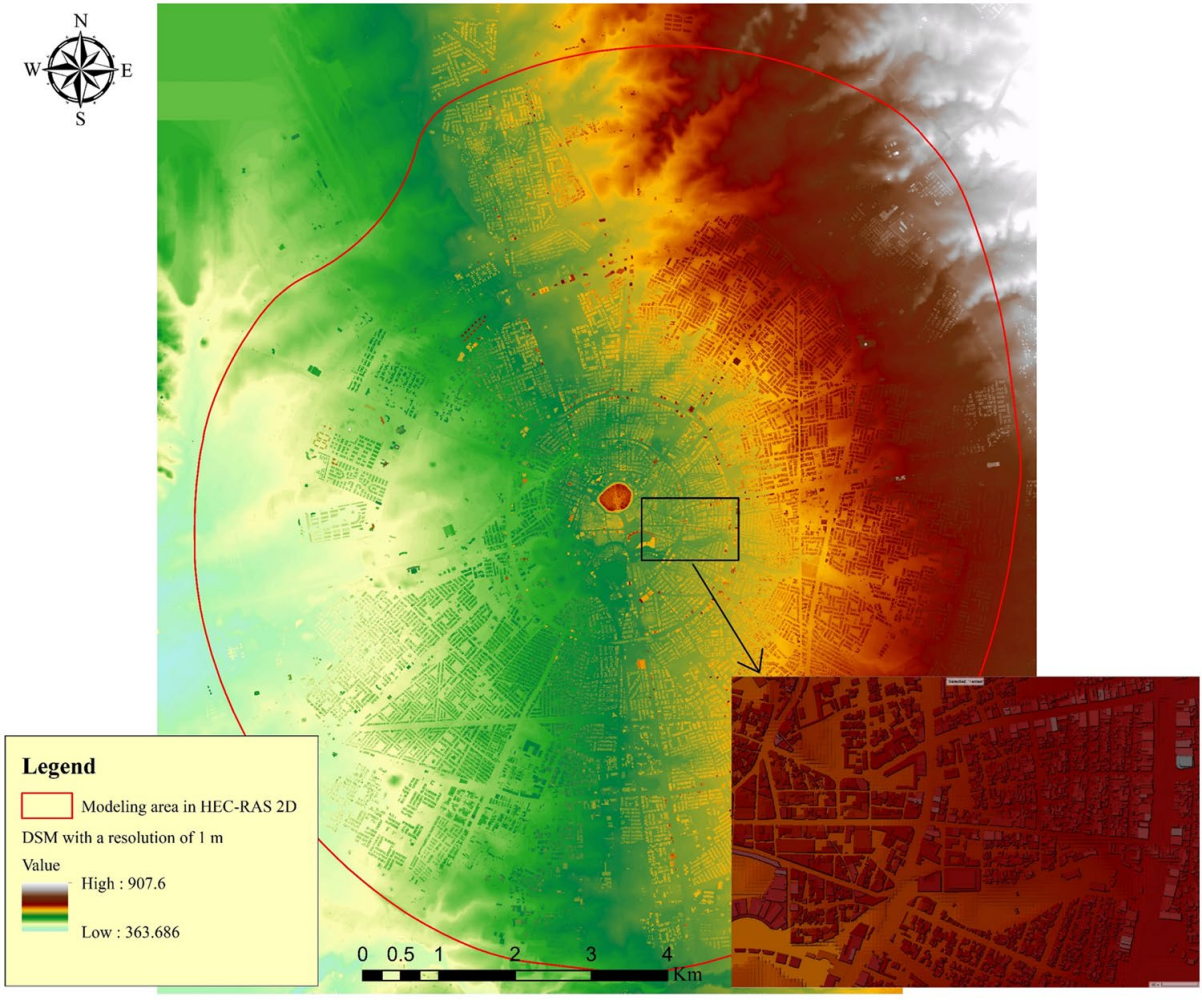
Digital Elevation Model (DEM) with 1 m resolution for modeling area (BB technique simulations) in HEC-RAS 2-D. This map is processed and generated in ArcGIS 10.7 software ( © ESRI, URL: https://www.esri.com/en-us/arcgis/products/index).

In the development and simulation of hydrological and hydrodynamic models, the availability of raw data is the most important factor to be considered. It was necessary to acquire a DEM from the Shuttle Radar Topography Mission (SRTM) (http://dwtkns.com/srtm30m/) with a resolution of one arc-second (30 m) in order to delineate the watershed boundary of the study area (Fig. 2). Li and Wong^63^ conducted an analysis to determine how various DEM data sources may influence the outcomes of hydrologic applications. A DEM with a resolution of 10 m was created using the point clouds of the LIDAR images (the data source was the Erbil municipality, the work date back to 2010). Then, a Feature Manipulation Engine (FME) was used to generate another DEM over the study area, from the layout map of the city, the heights of the buildings were extracted to create a building layer in three dimensions (3D). From the buildings, a raster with spatial resolution of 1 m was created. After resampling the DEM of the city (only the area inside 120 m ring road) from 10 to 1 m, it was then overlaid on the raster accompanied by the buildings. Raster calculation with maximum operation was applied to create a DEM of the city with a resolution of 1 m (Fig. 3).

**Figure 4:**
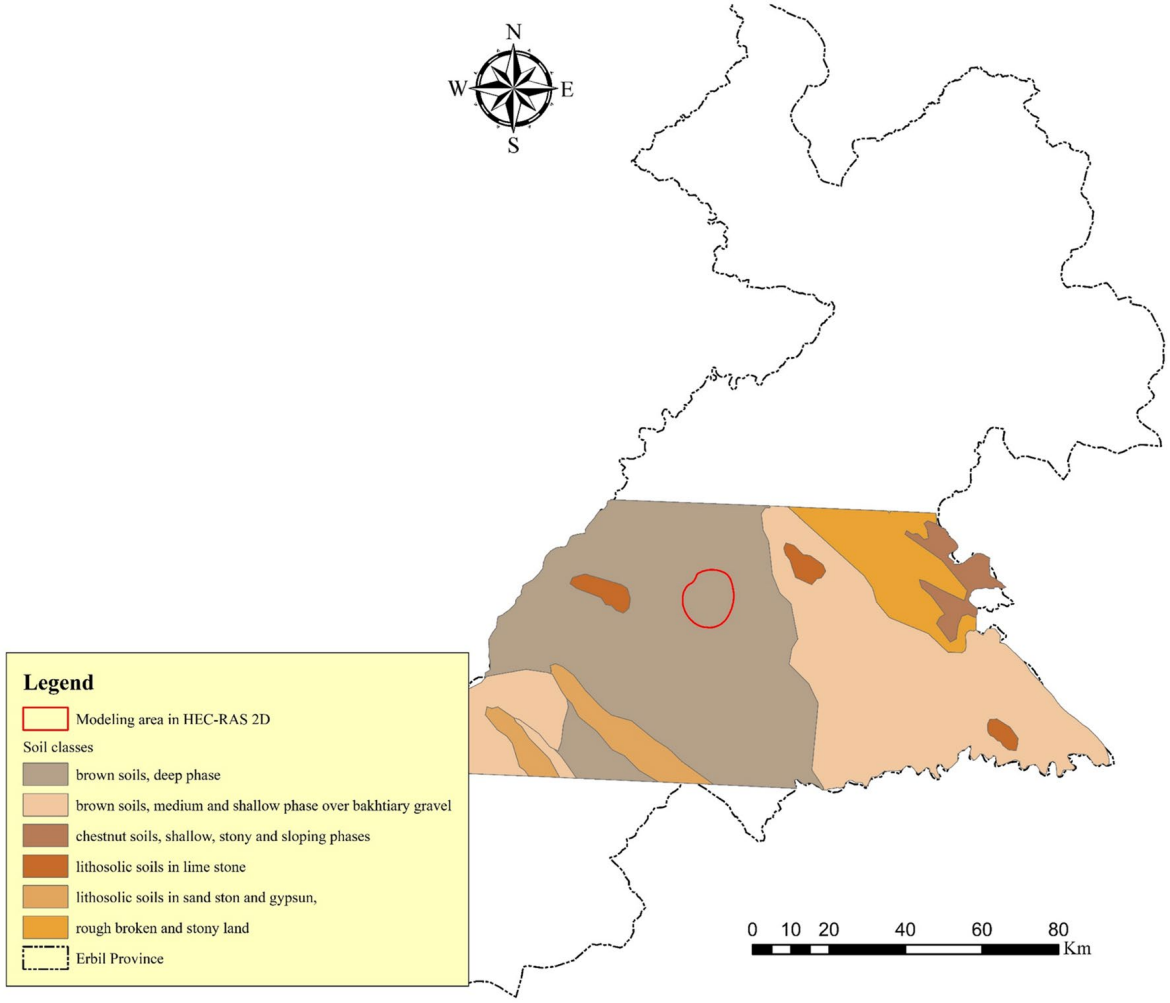
The soil classes in the study area. This map is processed and generated in ArcGIS 10.7 software ( © ESRI, URL: https://www.esri.com/en-us/arcgis/products/index).

The Food and Agriculture Organization of the United Nations (FAO) prepared a digital soil map of Iraq, from which a soil map of the studied area was extracted. Brown soils with deep phases have dominated the soil profile in the area under consideration for 2-D hydrodynamic modeling (Fig. 4). The Natural Resource Conservation Service (NRCS) divided soils into four Hydrologic Soil Groups (HSG) based on their runoff potential^64^. A, B, C, and D are the four HSGs, where A has the least potential for runoff and D have the most. The study area for modelling in HEC-RAS 2-D is in group C^62^.

**Figure 5:**
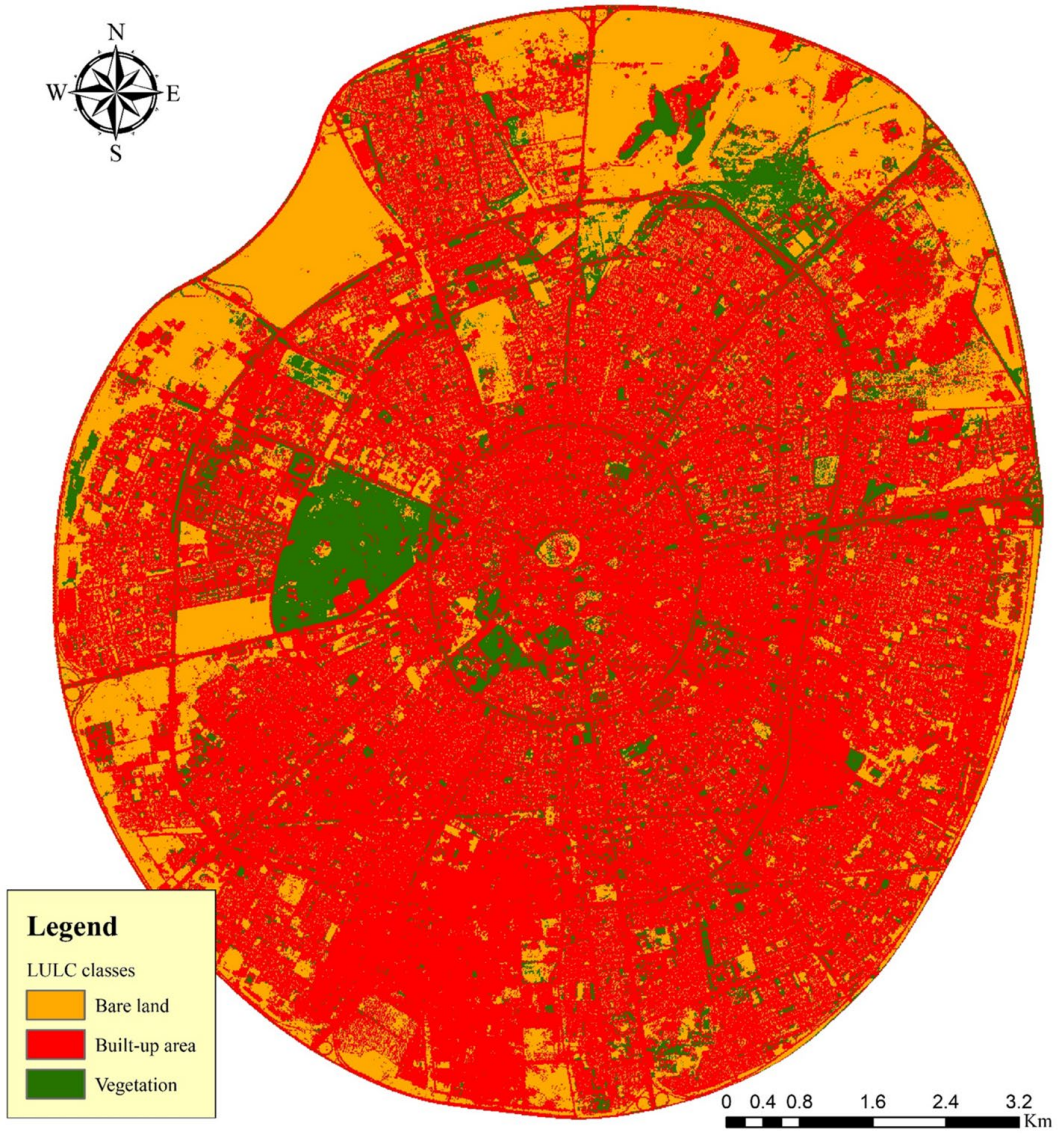
Land use land cover classes inside the modeling area in HEC-RAS 2-D. This map is processed and generated in ArcGIS 10.7 software ( © ESRI, URL: https://www.esri.com/en-us/arcgis/products/index).

The LULC map was created using data collected through remote sensing. When cloudy coverage is less than 5%, a satellite image from Sentinel-2B (2017–present) was downloaded from the Copernicus open access hub platform (https://scihub.copernicus.eu/), which is operated by the European Space Agency. The image was captured by a satellite on September 13, 2021. Regarding the LULC classification, the study area was divided into three categories: built-up (which included residential, industrial, commercial, local streets, roads, and other urban areas); bare land (which included uncovered soils, unused areas, and dry river beds); and vegetation (including forests, orchards, vegetable fields, parks, lawns, shrubs, and others) (Fig. 5). Approximately 66.36 km^2^ (64.82%) of the total study area is covered by built-up areas, while 25.038 km^2^ (24.45%) and 10.97 km^2^ (10.71%) respectively are covered by bare land and vegetated areas.

### HEC-RAS 2-D model setup

Water flow through natural rivers and other channels is modelled using HEC-RAS 6.1, an open-source software developed by the U.S. Army Corps of Engineers (USACE) to simulate hydrodynamics of water flow. One-dimensional 1-D steady and unsteady flow modeling, as well as two-dimensional (2-D) unsteady flow modeling, as well as combined one-dimensional and two-dimensional 1-D/2-D unsteady flow routing, sediment transport/mobile bed computations, and water temperature/water quality modeling are all possible with this software^65,66^. The Digital Terrain Model (DTM) is responsible for inducing the majority of the topographic characteristics of the study into the HEC RAS 2-D model. Using a DEM as an input file, HEC RAS 2-D is capable of creating a DTM within the program. Specifically, a DEM plus buildings with a resolution of 1 m was prepared for use in the generation of the study area's DTM in this case. In the geometric data editor of the software, a 2-D flow area describing the boundary of the assumed flood domain is created by drawing a polygon with the orthophoto as the background layer. Afterwards, an automatically generated computational mesh within the boundary layer is created with a 10 × 10 m cell size. This results in a total of 1,022,897 grid cells with an average size of 100 m^2^. To demonstrate the effects of computational cell size on the model outputs and model run time, two more mesh configurations with 8 × 8 and 20 × 20 m are examined. The total number of grid cells in these mesh configurations is 1,598,552 (average cell size = 64 m^2^), and 255,501 (average cell size = 400 m^2^), respectively. The four building representation techniques that are commonly used to model built-up area flooding in hydrodynamic numerical models are the BB, BR, Building Hole technique (BH), and Building Porosity technique (BP)^67–71^. In the HEC-RAS 2-D, both BB and BR are applicable. BB technique means increasing the ground elevation of building units by modifying distributed ground elevation data, by configuring the buildings to a real height or a sufficiently high artificial elevation value to ensure no water flows over the buildings. Here, the entire simulated flow area should be meshed as a unified grid, so water flows around buildings. In BR, the modeler may give each grid a different Manning coefficient. High Manning coefficients result in low water flow velocity. In this method, the simulated building areas are given a high Manning n value to increase friction. In other simulated areas, a low value represents the real land cover. Due to the high friction coefficient assigned to the building units, water flows slowly over them but behaves as if there is a high resistance against flow. A detailed description can be found in^43^.

The model's other parameters, which include 2-D surface roughness, curve number for LULC classes, boundary conditions, and rainfall data, are also required. Table 1 categorizes the LULC classes, Erbil City center is a densely urbanized area with predominant brown soil and deep phase. Thus, based on imperviousness and soil type, three different Manning's n are selected for the 2-D area^72^; see Table 2. Defining boundary conditions within the HEC RAS 2-D flow area is required in order to run the 2-D model simulations. Boundary conditions for defined 2-D flow areas can include a variety of features such as flow and stage hydrographs, normal depths, rating curves and precipitation boundary conditions, among other things. In the current study, only 23 outlets are defined as boundary conditions with normal depth. A time series of rainfall is used as meteorological data in the unsteady flow section for each storm event, and a simulation run is carried out to generate flood inundation for each storm event.

**Table 1:** Curve number (CN) values^73^.

LULC classification	Curve number for hydrologic soil group			
	A	B	C	D
Built-up area	77	85	90	92
Bare land	68	79	86	89
Vegetation	43	65	76	82

**Table 2:** Average values of Manning’s roughness coefficient based on different LULC classes^72^.

LULC classification	Manning’s n value
Built-up area	0.013
Bare land	0.03
Vegetation	0.025

**Figure 6:**
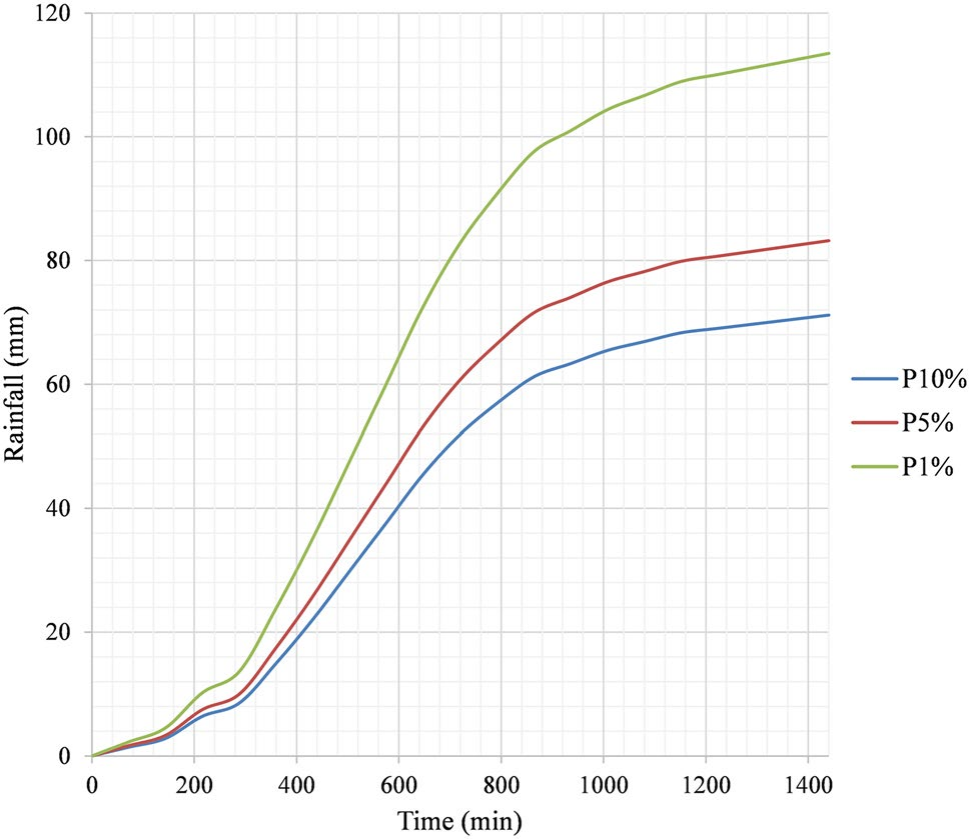
Temporal distribution of rainfall for three probability distributions of 10, 5, and 1%.

**Figure 7:**
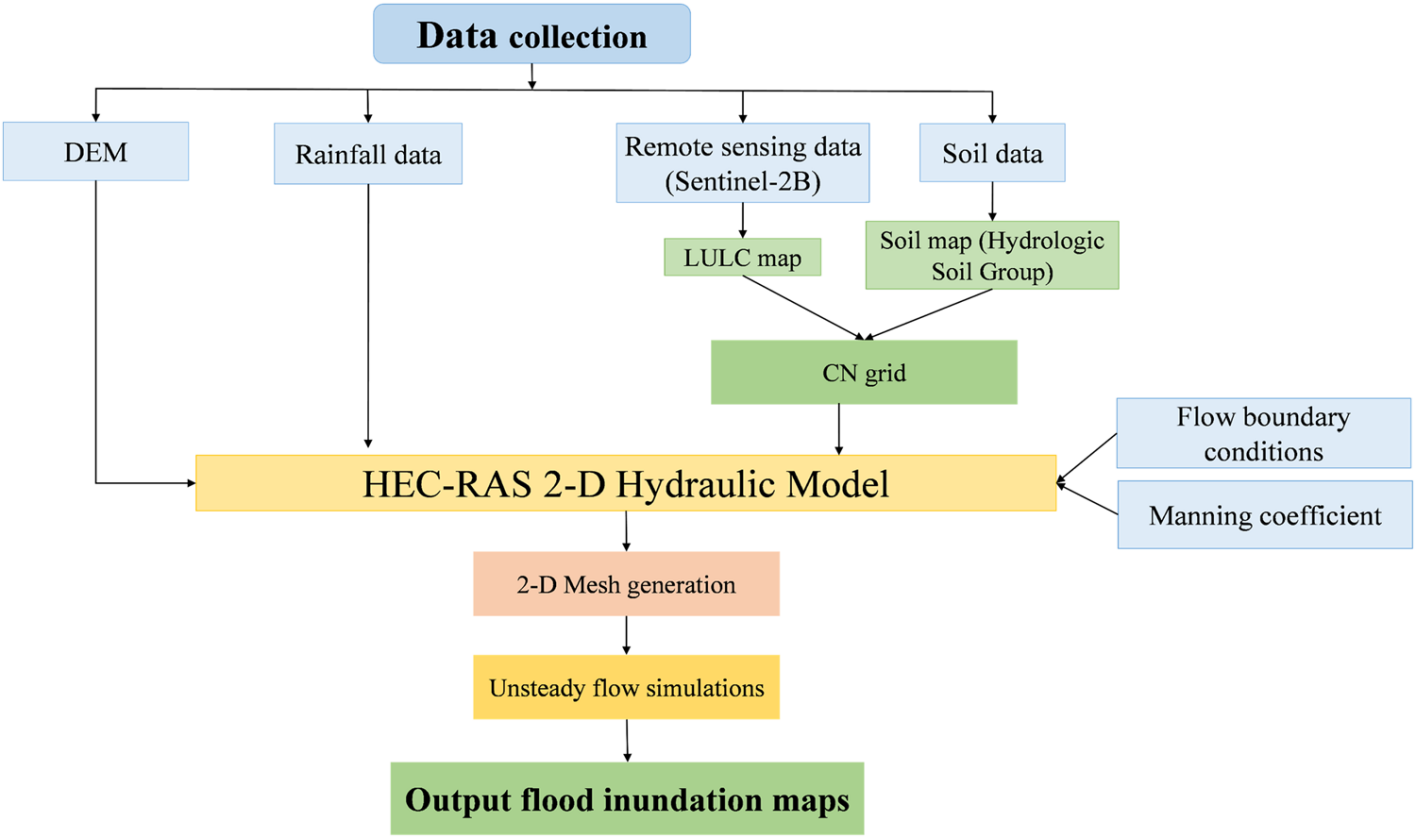
A flowchart illustrating the steps involved in developing a HEC-RAS 2-D model.

Analyzed long-term series of maximum daily rainfall data were used in this study and within them, a theoretical probability distribution of 10, 5, and 1%, which is equal to 71.16, 83.21, and 113.49 mm^47^ (see Fig. 6). The method that was used to prepare the temporal rainfall distribution was based on Huff's second quartile^74^. The reasons for using this method are explained in^47^. Uniform rainfall distribution in space is considered. Unsteady flow routing can be done two-dimensionally with either the Shallow Water Equations (SWE) or the Diffusion Wave equations (DWE). To solve the flow over the computational mesh using HEC-RAS, three sets of equations are available: the Diffusion Wave equations; the Shallow Water Equations (SWE-ELM, or Shallow Water Equations, Eulerian–Lagrangian Method) original equations; and a more momentum-conserving Shallow Water Equations solution (SWE-EM, which stands for Shallow Water Equations, Eulerian Method)^66^. In the present study, the original (SWE-ELM) was considered for HEC-RAS 2-D. The flowchart shown in Fig. 7 illustrates the process of developing a HEC-RAS 2-D model.

## Results and discussion

### Analysis of different mesh resolutions and building representation techniques

This study compared two different building representation techniques as well as the outputs of various simulations using fine and coarse grid sizes in order to analyze the results. For modelling floods in urban areas, a fine 2-D grid, typically less than 5 m, is required to accurately simulate the flow on streets, intersections, around houses and buildings^75^. In the beginning, we focused on the BB technique to determine whether or not the prepared DEM was suitable for such numerical simulation. Because we believe that the quality of the DEM that was prepared is at an acceptable level. In this case, we were looking for the answer to the question of whether or not this model would provide us with results that were satisfactory. In our study, we used three different grid sizes, starting at 8 m and the others at 10 and 20 m. In fact, because of the size of the area considered in the modeling in HEC-RAS 2-D, it was difficult to simulate the flow using a grid size smaller than 8 m. The fact should be noted, however, that shrinking the size of hydrodynamic grid cells results in denser mesh and therefore requires a longer run-time to complete^76–78^, necessitating a trade-off between simulation accuracy and calculation time. In a study by^79^, two novel methodologies for reducing the processing time of 2-D large-scale flood simulations are compared in order to evaluate their merits and drawbacks and provide advice for their effective application. Specifically, Yalcin^80^ stated that mesh resolutions created at the basin scale with a size greater than 10 × 10 m, referred to as a "coarse grid," cause significant errors in the estimated inundation extent because they are unable to capture rapid changes in the terrain geometry.

**Figure 8:**
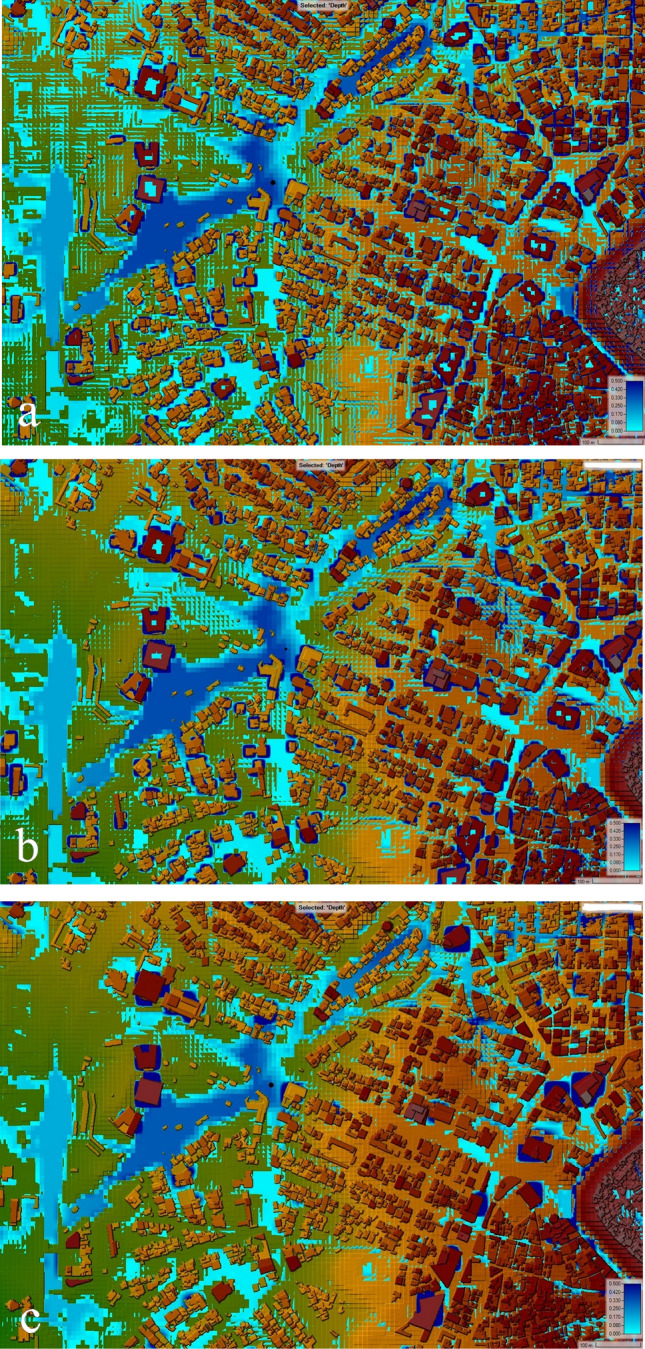
Analysis of the numerical simulation using different mesh resolutions with the BB technique: (**a**) Grid size 8 × 8 m. (**b**) Grid size 10 × 10 m. **c** Grid size 20 × 20 m. These maps were processed and generated in ArcGIS 10.7 software ( © ESRI, URL: https://www.esri.com/en-us/arcgis/products/index).

**Figure 9:**
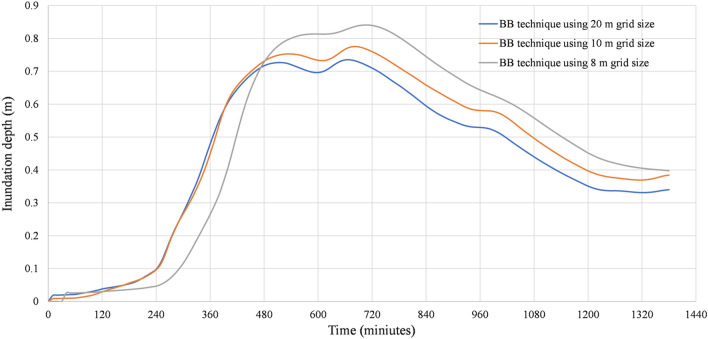
Comparison of inundation depth between BB technique simulations.

To shed light on the role of grid size in numerical simulation, multiple simulations have been done. When the BB technique was simulated using cell sizes of 8, 10, and 20 m, the results of the simulations revealed an error in the water depth calculation. In front of the buildings, a large amount of water has accumulated. We believe that this error is due to the disparity between the dimensions of the street and the size of the cell (See Fig. 8a, b, and c). When more precise cell sizes are utilized, it is anticipated that the error will decrease. Our attention was drawn to something, water propagation and water depth in front of buildings can be improved if the grid size is reduced. The DEM and mesh resolutions are the most significant elements to consider when running simulations of water level dynamics using the BB technique. On the other hand, the floodplain roughness coefficient is a crucial consideration for mapping the extent of floodplain. According to Alipour, et al.^44^, the resolution of the DEM is the most essential factor across all scales for the simulation of the dynamics of water levels. In their research on the flood inundation simulation of the Mahanadi River in Odisha, Surwase, et al.^81^ found that the HEC-RAS model is sensitive to the manning roughness coefficient. Figures 8a, b, and c show a random location within the 2-D geometry area to demonstrate flow propagation. Figure 9 illustrates the levels to which the inundation depth is sensitive to the grid size used in the BB technique simulations. Because these simulations give a false impression of the extent and depth of the flood, they should be ignored in most cases. Because of the capabilities of the computer processor, it was not possible to reduce the cell size to less than 8 m. A mesh of this size would be unable to accurately simulate the abrupt change in the terrain. As a result, we came to the conclusion that the size of the 2-D flow area needed to be reduced in order for us to be able to model the area using a grid size of 2 × 2 m or even less than 2 m.

This is not the end of the story because it is the responsibility of the researchers to investigate all possible avenues so that they can eventually present a conclusion to society. Because we were interested in determining the effect of urban flooding inside of the 120 m ring road in Erbil, we made the decision to model this zone using a different method, which is known as the BR technique. Previously, Mustafa and Szydłowski^43^ have validated this method by applying it on a small scale to a physical model of the Toce river. The Manning coefficient is a coefficient describing the roughness or friction of a surface in the field of flow, that estimates the average flow velocity. In the BR technique, Manning coefficient values (such as 10 m^−1/3^ s) are assigned to all the mesh regions that represent the building blocks (which here are known as user-defined polygons) to examine the resistance against the flow. When compared to the BB techniques, the results demonstrated a more accurate estimation of the water depth within the same grid size and geometry area. In point of fact, the BR method allows for the possibility of water entering the houses and buildings, whereas the BB method ensures that this will not occur because the building is modelled in the form of a block. In terms of the calculation of water depth, BR may give better results, which is something we could say in response to the question of which method is more realistic. If a high-resolution DEM is used in the modeling and simulation process, it is expected that the BB technique will give more accurate results about the flood extent and the most vulnerable areas.

### Assessments of the areas affected by flooding

We came to the realization that BR is an effective technique for conducting analysis on floods in the study area, obviously, we reached this conclusion due to the lack of reliable data. Hence, the analyses presented are based on the BR technique with a 10 m grid size. Moving on, it is clear that we made some assumptions in order to develop the model for the area considered in HEC-RAS 2-D, one of which was that there is no inflow to the area at all, which is not true because there is inflow to the 2-D area through main roads and some underground box culverts that cross the 120 m ring road. However, because there is no recorded data regarding the flow, we made the decision to keep the boundary closed. Despite this, we are aware that this assumption has an impact on the results; however, by constructing some retention basins, we can prevent flow from the outside of the studied area into the inside. Beyond assumptions, we can confidently state that our modeling is accurate to a significant degree because when comparing our results with those areas known as prone areas, the accumulation of water in the HEC-RAS 2-D is calculated.

**Figure 10:**
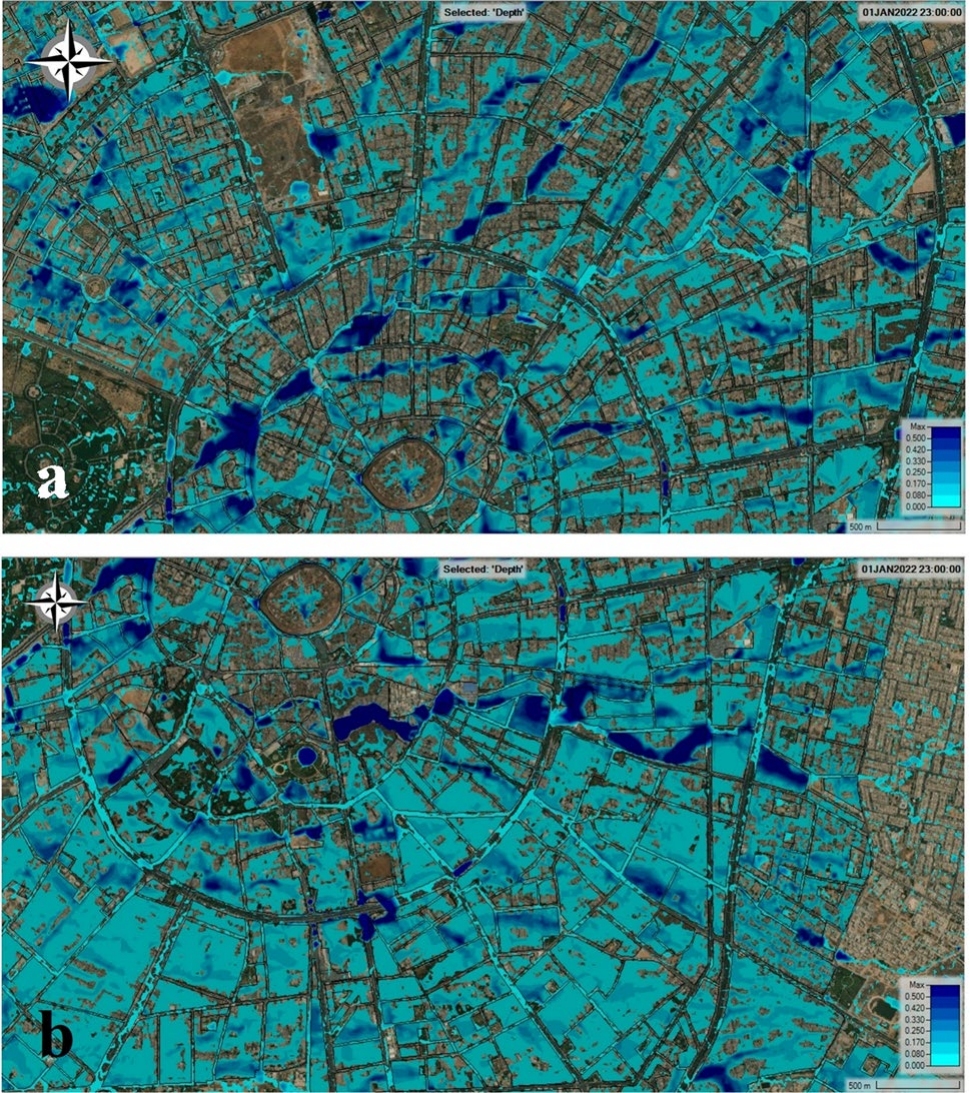
Results of the numerical simulation using BR technique with grid size 10 × 10 m: (**a**) upper part of the studied area. (**b**) the lower part of the studied area. Base imagery sources: Esri, DigitalGlobe, GeoEye, i-cubed, USDA FSA, USGS, AEX, Getmapping, Aerogrid, IGN, IGP, swisstopo, and the GIS User Community. These maps were processed and generated in HEC-RAS 6.1 software (https://www.hec.usace.army.mil/software/default.aspx).

Using two orthogonal components of the flow, 2-D surface flow modeling attempts to model the propagation of overland flow. It is possible to predict the maximum inundation area and the flow dynamics, such as water depth and velocity, using 2-D modeling of the surface flow^82^. When we look at the extent of the flood, it is immediately apparent that water has accumulated in low-lying areas. Flood accumulation areas are often identified by their low elevation, which is a good indicator of their risk of flooding^28^. Furthermore, despite changes in the city's layout, the flow movement continues to follow the same path that it did a hundred or even a 1000 years ago. For example, the source of accumulated water in both Tayrawa street, particularly Tayrawa Bazar, and the main gate of the council of ministers on 30 m street is coming from the northeastern areas of the studied areas, such as the North industrial area and the area near the Family Mall shopping center, according to the findings (Fig. 10a). The source of the flow that causes problems in Setaqan quarter on 60 m street, then in Saydawa quarter near the old Saydawa bridge, and near Nishtiman Bazar, before finally passing through the city via an underground box culvert, is located in the eastern part of the study area, and it comes from the Havalan quarter (Fig. 10b). When the volume of flow exceeds the capacity of the box culvert, overflows occur in this zone. This event occurs after heavy rainfalls for a variety of reasons, including the flow from the surrounding area being accumulated over there, the area serving as a flow route, the stormwater system at this location appearing to be inadequate, old and finally, the accumulation of debris inside inlets, manholes, and box culverts, which prevents the system from functioning properly.

The inundation depth (m) maps that were generated over the course of the simulation run period are used as criteria to describe the extent of the inundated areas. Although the area of flood inundation and its depth are the most critical parameters, particularly when mapping flood hazards^83^. We chose to focus on the extent and prone areas of flooding rather than the depth of inundation because the surface flow is all that is calculated during simulations. For the reason that some of the surface runoff will pass through the existing stormwater system. In addition, we performed three separate simulations based on a theoretical probability distribution of 10, 5, and 1%, which is equivalent to 71.16, 83.21, and 113.49 mm, respectively (the duration of the rainfall event was 24 h).

**Figure 11:**
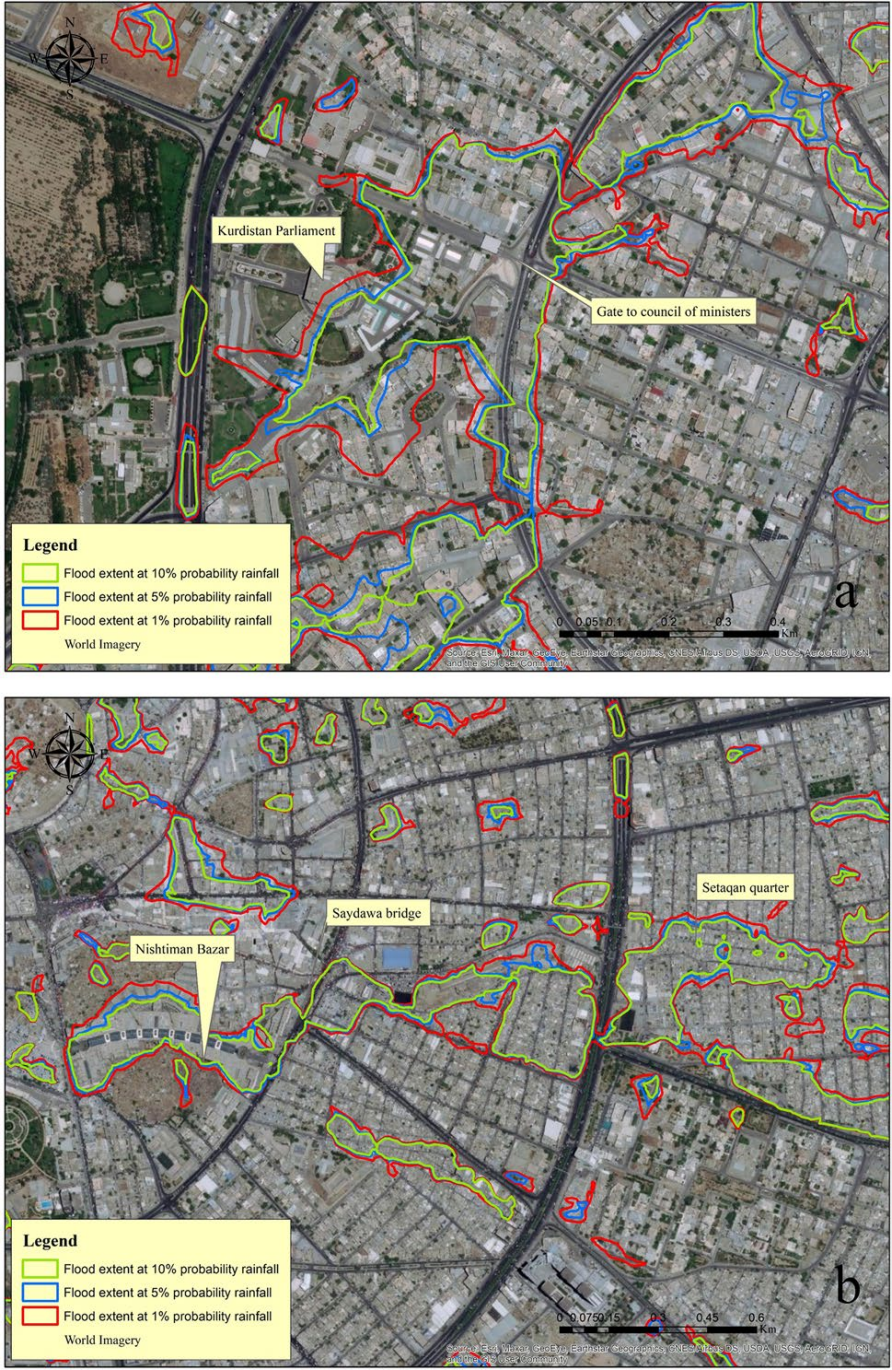
Comparison of the development of flooded areas based on a different rainfall simulation: (**a**) Gate to council of ministers. (**b**) Nishtman Bazar, Saydawa quarter and Setaqan quarter. Base imagery sources: Esri, DigitalGlobe, GeoEye, i-cubed, USDA FSA, USGS, AEX, Getmapping, Aerogrid, IGN, IGP, swisstopo, and the GIS User Community. These maps were processed and generated in ArcGIS 10.7 software ( © ESRI, URL: https://www.esri.com/en-us/arcgis/products/index).

**Figure 12:**
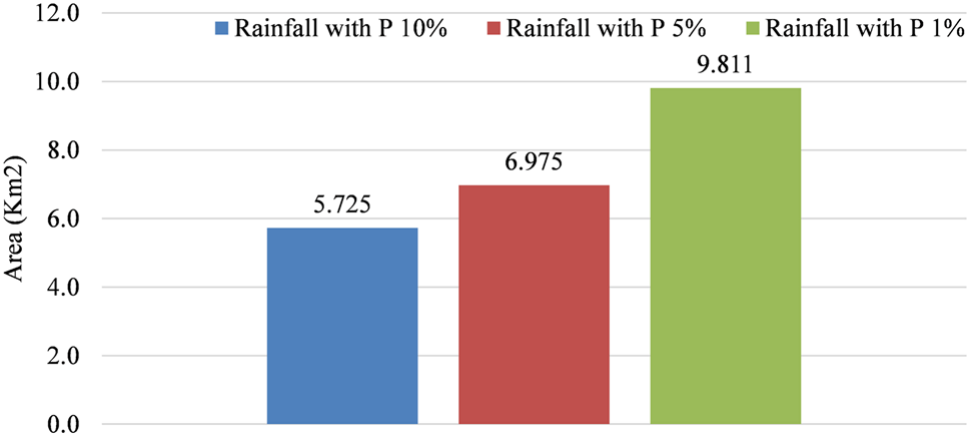
The evolution of inundated areas.

Here, in order to delineate flood extent in the studied area, we generated a vector layer using water depth maps at a value of 0.2 m. This means that the program will develop a vector layer for all areas where the water depth is equal to and/or greater than 0.2 m. This was done so that we could determine the extent of the flooding. After that, the vector layer was saved by being transformed into a shapefile. The vector layer was then sorted using the attribute tables, and areas smaller than 500 m^2^ were ignored. As can be seen in Fig. 11a and b, the amount of rainfall that occurs will cause the flooded area to expand, and this expansion will be proportional to the amount of rainfall that occurs. When the simulation was performed with a rainfall probability of 10%, an area of about 5.725 km^2^ was affected by flooding. The size of this affected area increased by about 21.83%, which is equivalent to 1.25 km^2^, when the rainfall probability was increased to 5%. In addition to this, when we simulated this time with a rainfall probability of 1%, it resulted in a 71.37% increase in the magnitude of the flood extent (Fig. 12). In point of fact, the areas of the studied site that experience water levels that are higher than 0.2 m in the case of P 1% rainfall have an area that is approximately equivalent to 10% (9.811 km^2^) of the total area (102.38 km^2^). It is concerning that the majority of the areas that flood or accumulate water as a result of rainfall are public streets, commercial districts, and residential areas. As a direct consequence of this, both citizens and businesses experience a loss of life as well as property. It is a common observation in Erbil that rain that falls in a concentrated burst over a short period of time causes the water level in the streets to rise, which in turn has a significant effect on the flow of traffic. Other flood hazard parameters, such as velocity, could not be developed by us because of the quality of the data that was available and the level of uncertainty that we produced during the modelling process. It was mentioned that only 10.71% of the studied area is covered by vegetation, and the majority of this vegetation can be found in two large areas in the middle of the city. Because the permeable areas were not distributed well, there was less infiltration and more runoff as a result.

**Figure 13:**
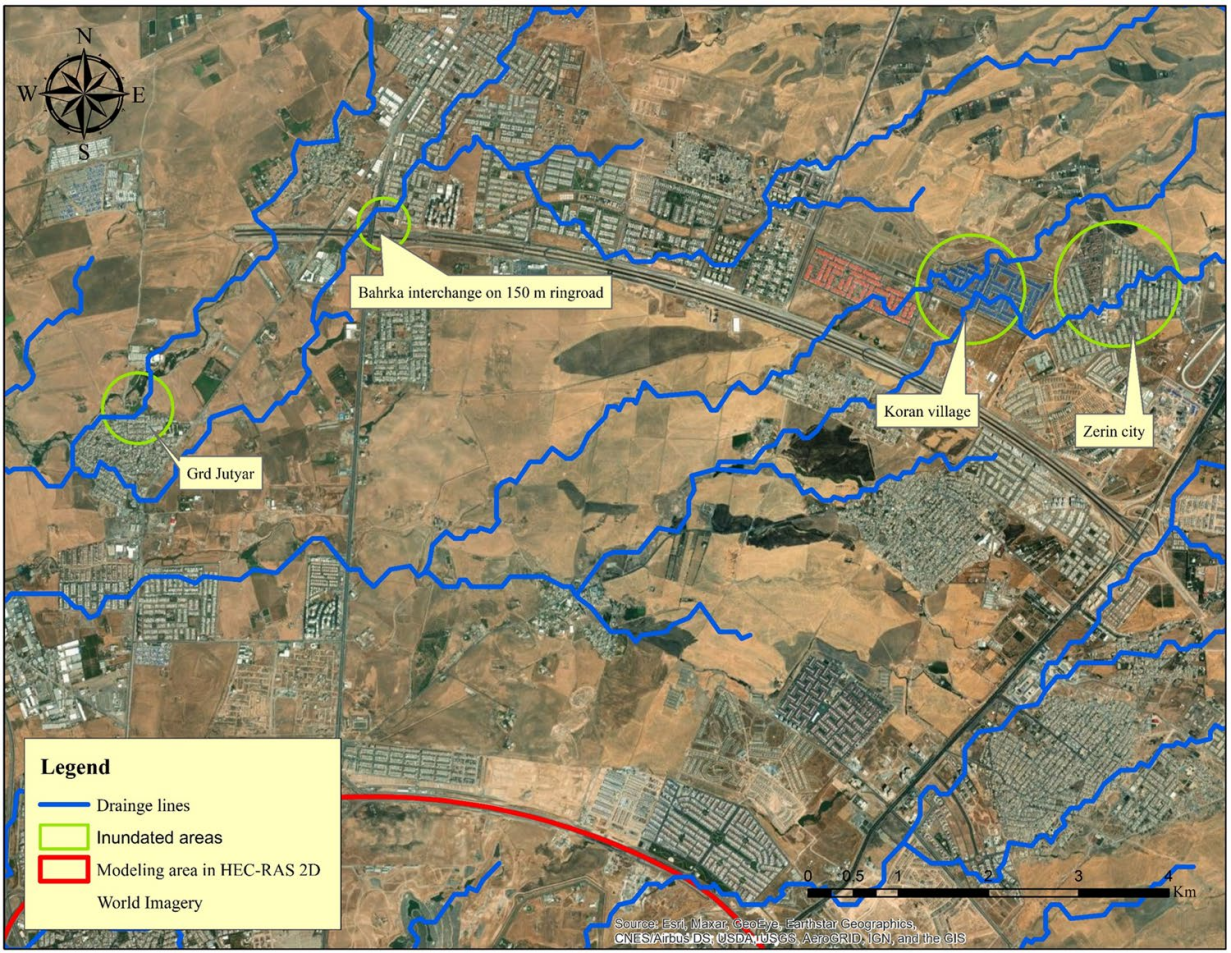
Some inundated areas in Erbil during the storm event of October 30 and December 17, 2021. Base imagery sources: Esri, DigitalGlobe, GeoEye, i-cubed, USDA FSA, USGS, AEX, Getmapping, Aerogrid, IGN, IGP, swisstopo, and the GIS User Community. This map is processed and generated in ArcGIS 10.7 software ( © ESRI, URL: https://www.esri.com/en-us/arcgis/products/index).

Observing two extraordinary events that occurred at the end of 2021, authorities in Erbil revealed that the flooded area is located on the route of natural and seasonal streams, which occurred outside the area considered in the HEC-RAS 2-D modeling in the current study. The expansion of the city has resulted in the construction of numerous residential and commercial buildings, as well as industrial and manufacturing facilities, along the path of natural flow routes. In this section, we evaluated the areas that were at risk of flooding outside of the 120 m ring road by using the DEM with a resolution of 30 m to assess flood basins that had been delineated. The route of the natural streams can clearly be seen in Fig. 13, which shows that both Zerin city and Korean village are located along the route of the natural streams. According to a media statement issued by the governor of Erbil on October 30, 2021, an amount of 55 mm of rainfall was recorded in the upper part of the city in just two hours on that day. In the northeast, where there is higher topography known as the Tarin heights, crazy flow can flow to the prone areas.

The same can be said for the village of Grd Jutiar, which is located directly adjacent to the natural canal. In fact, on that particular day, the canal was unable to handle the volume of water flowing through it. As a result of this, the overflow of canal banks occurred somewhere near the north side of the village, resulting in the catastrophic event. The first phase of a 150 m ring road connecting the main Shaqlawa road with the main Bahrka road was completed in 2021. In an interview with Rudaw TV^84^, the project manager for the 150 m ring road stated that the culverts and box culverts that cross the ring road and run along the ring road were only intended to handle stormwater from the surrounding areas and household waters, not the large amounts of water that flow from the natural basin. Erbil city has expanded toward mainly the Northeast and East areas, but detailed hydrologic studies are absent. Many stormwater and sewer pipes in local areas throughout Erbil have been built without taking the dimensions of water pathways and streams into consideration. It is now possible to experience flash flooding in areas where urban and infrastructure development has taken place.

**Figure 14:**
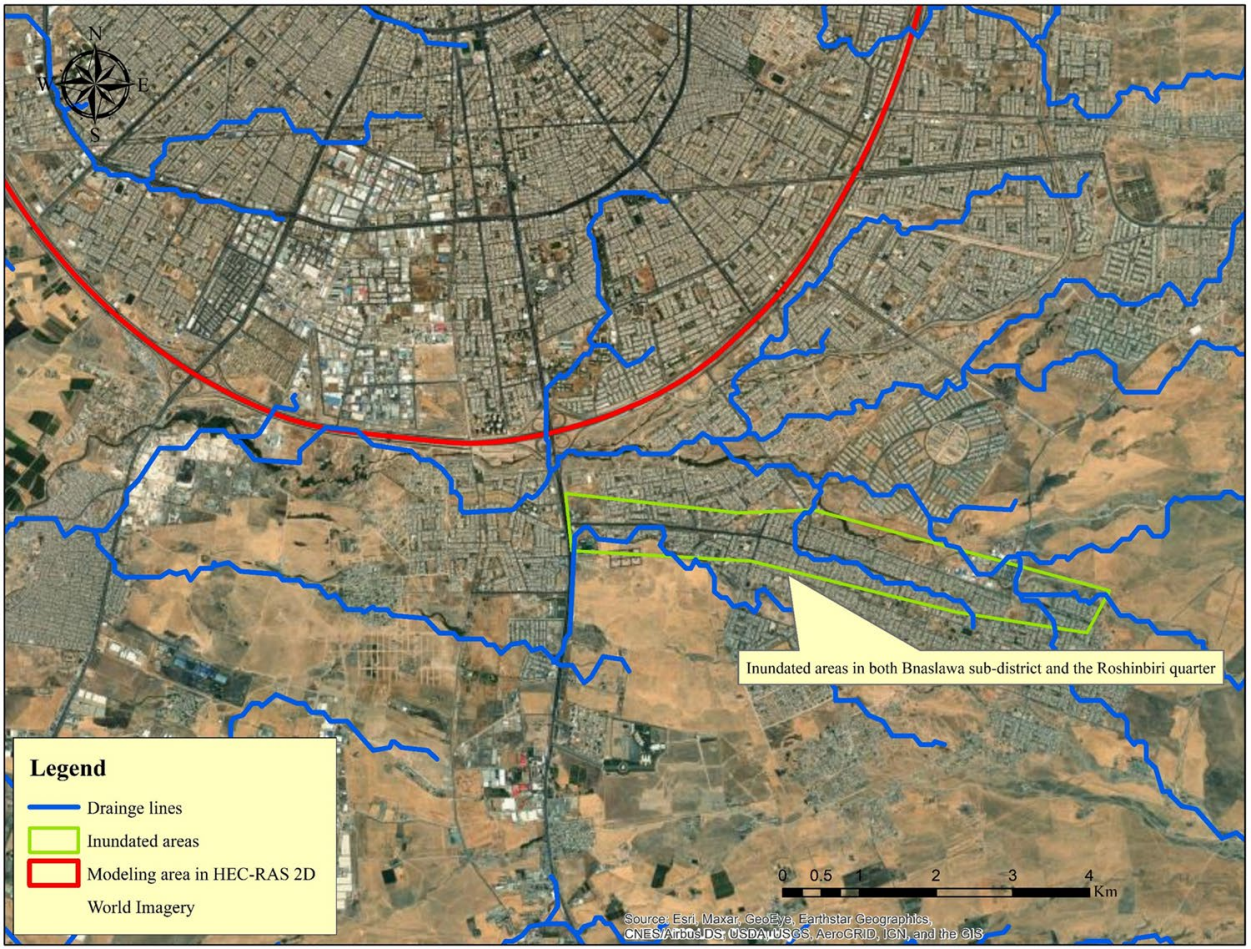
Bnaslawa sub-district and the Roshinbiri quarter were inundated in Erbil during the storm event of December 17, 2021. Base imagery sources: Esri, DigitalGlobe, GeoEye, i-cubed, USDA FSA, USGS, AEX, Getmapping, Aerogrid, IGN, IGP, swisstopo, and the GIS User Community. This map is processed and generated in ArcGIS 10.7 software ( © ESRI, URL: https://www.esri.com/en-us/arcgis/products/index).

Flooding in Erbil ranges from minor incidents, such as inundation of streets and then water entering some houses, to major incidents, such as large areas of the city being submerged for several hours at a time. The event, which took place on December 17, 2021, was primarily focused on the Southeast of the city, specifically the Bnaslawa sub-district and the Roshinbiri quarter (Fig. 14). In reality, this was a catastrophic event that was unprecedented in Erbil's history. Because 12 people died, one of them was a 10-month-old child whose body was found approximately 60 days after the event and more than 15 km away from his house. A large number of small-scale local problems are common in most developing cities around the world, primarily because their stormwater systems do not have enough capacity to handle heavy rainstorms^85^. Greater flooding risk exists in areas that are close to the main channel and accumulation path^86^. Poorly designed highways that do not take into account natural drainage patterns can lead to increased runoff and flooding during heavy rainfall events^87,88^. There were various contributing factors to this catastrophic event, the first of which was a reduction in the cross-section of streams, with their bed elevations approaching the elevation of the street in some places. The reason for this is that these seasonal streams have not been cleaned in a long time and have become clogged with river sediments and debris. In addition, a large number of quarries are operating on the river bed on the upstream side of the developed area (Bnaslawa sub-district) to extract sub-base or other materials. According to the locals' speech, those quarries created a dam-like barrier, which collected a large volume of water on that particular day before being breached, causing the flow to become increasingly erratic. As we can clearly see, a large number of residential areas have been constructed directly adjacent to stream routes, indicating that this type of event is predictable.

**Figure 15:**
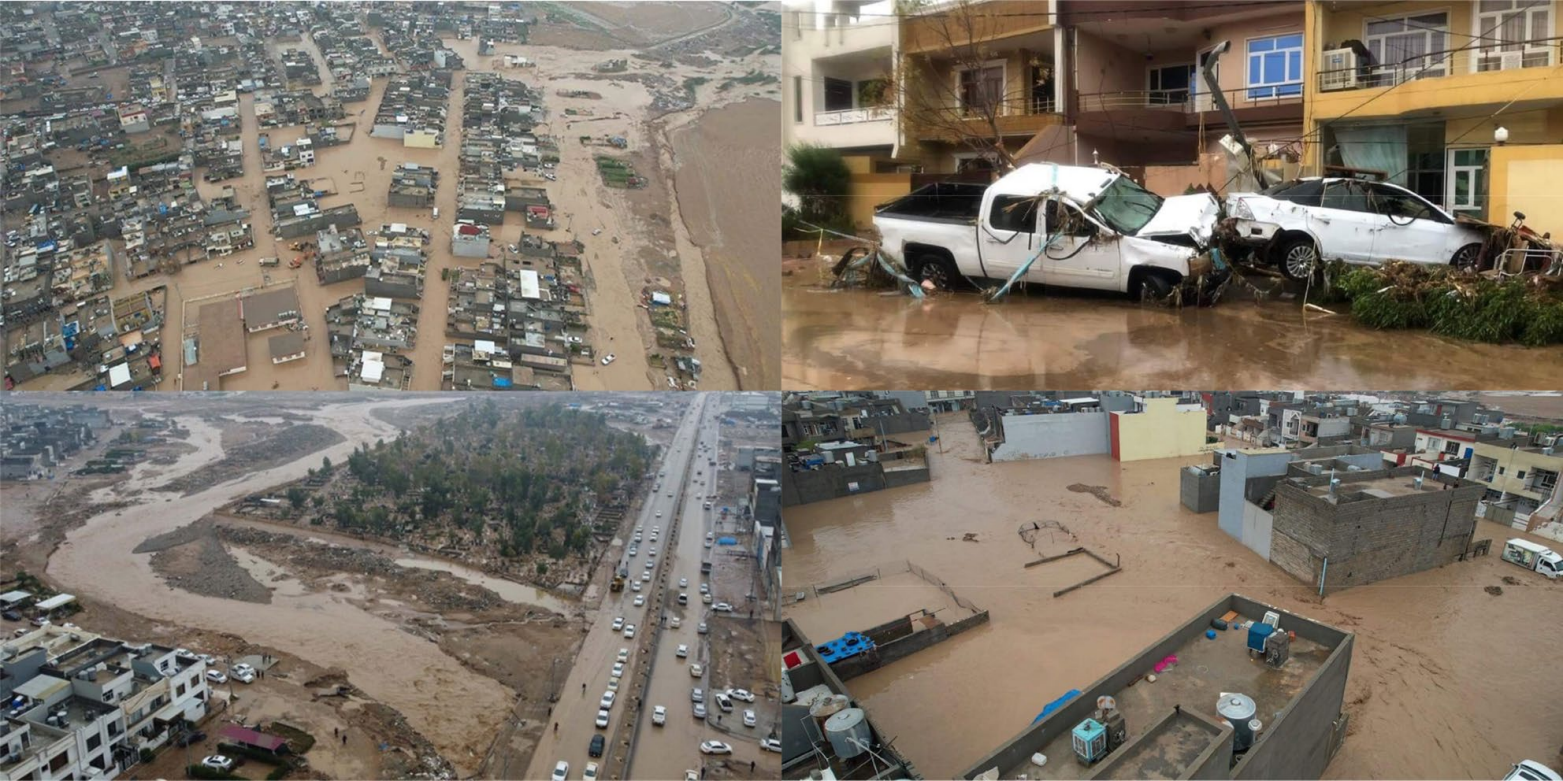
Flooding had devastating consequences in Erbil in two separate events at the end of 2021.

The floods in Erbil cause broad damage on several levels (Fig. 15). On a human level, floods claim a number of lives. Economically, urban areas are the most frequently affected, and this is especially true in the most severe events. Environmental consequences are big, and we are seeing deterioration of the natural environment, as well as the production of debris and wreckage, which is being spread over drainage lines and residential areas. There are several different approaches that can be taken to mitigate the consequences of flooding and heavy rainfall. One of the strategies is to make individuals aware of locations that are at risk of flooding and to warn them accordingly. For example in Brazil, a Decision Support System (DSS) coupled with a flood alert web application was developed and evaluated in the Prosa Basin (Midwestern Brazil) to send early flood warning messages to ordinary people^89^ . The basis of the flood alarm system was the Hydrologic Modeling System (HEC-HMS) and River Analysis System (HEC-RAS) of the Hydrologic Engineering Center.

## Recommendations, suggestions and the future scope

In accordance with the findings of this study, the recommendations made priorities the implementation of a plan for flood hazard prevention and the protection of the city and its infrastructure through the maintenance of existing floodwater drainage facilities. The result of this study highlights the necessity of establishing hydrodynamic facilities for the control, obstruction, and divergence of the floods of drainage lines, and the specific recommendations are as follows:

- The adoption of a plan to harvest water in the northeast and east of the city would make a significant contribution to the provision of water for people and farms. It would also aid in the process of afforestation, which slows down floods and allows them to feed the underground reservoirs. At the same time, it would reduce a portion of the wasting floods that are directed towards the city center. Moreover, in order to reduce the craziness of the flow and delay the flow into the city.
- Cleaning and maintaining the width of seasonal streams and drainage lines, which are located in various parts of the city, especially those located in the east, the northeast and the southeast. Also necessary is the refinement of the slope of the drainage channel in the area in order to increase the drainage velocity.
- A reserve for each stream must be established to prevent construction in drainage lines and flood catchments. This reserve must remain in place even after the proposed man-made channels have been constructed. A mandatory study of flood risks, including their peaks and catchments, must also be provided with each project and submitted to the appropriate authorities for consideration of approving the project.
- The stormwater management system should be changed/modified so that it can keep up with urban development, population growth, and climate change. Because there was no way to show that they were the same.
- Green roofs, rainwater harvesting systems in the household, permeable surfaces, swales, channels and rills, infiltration trenches, detention and infiltration basins, rain gardens, and retention ponds are all examples of Nature-Based Solutions (NBS) that can be used to improve the capacity of stormwater systems. Certainly, this will be after evaluating the outcomes of each solution using high-resolution models and selecting the most suitable option for the research area.
- The uneven distribution of the city's greenery has contributed to the problem of increased runoff. One way to lessen the impact of this is to reorganize the green spaces within the city layout. In this regard, precautions should be taken in areas that are prone to flooding.
- Local and regional authorities should develop flood control and protection legislation and regulations in order to save lives and property from the effects of flooding. Having such types of national laws in place is extremely important for managing flood risk and increasing future flood resilience in floodplains.
- The Storm Water Management Model (SWMM) or a coupled model can also be used to evaluate the efficiency of the current urban drainage system in Erbil city center, which will allow for a more accurate analysis of urban flooding. However, because there are currently no GIS-based stormwater lines in Erbil, a significant amount of effort may be required. The model can simulate storm events and the resulting flooding scenarios and shall help the authorities in the directorate of water and sewer/Erbil devise an efficient drainage system in the city. Filling in the gaps in the current study will result in a completely different understanding of the Erbil flooding process in the future, which will aid the authorities in developing a more comprehensive flood management and mitigation plan.

## Conclusions

Climate change, which causes and amplifies large-scale natural disasters, poses a threat to the city of Erbil. These dangers can sometimes have a negative impact on people's daily lives and activities in the city. Our previous paper clearly illustrates the magnitude of the flooding phenomenon that has occurred in this area. Flash floods in urban areas have been and will continue to be a major problem in many cities throughout the world, particularly in developing countries. The threat of flooding can be countered by the opportunity it provides to bring in more water. As a consequence of this, flood management is a component of water management, and it should be given particular consideration in the Kurdistan Region of Iraq, particularly in the city center of Erbil, where solutions to the problem of water scarcity are required as quickly as possible. Due to the rapid growth of Erbil as a city in a developing country without the necessary funds to expand and renovate their existing drainage systems, the situation continues to deteriorate further. The findings of this study tell us that the effects of flash flooding in Erbil in the areas shown are due to their location that is located on the waterways, and even the areas that were flooded in the first month of 2022 in the city center were waterways in the past. Therefore, we believe that serious and practical steps must be taken in order to lessen the effects of floods, which will allow for runoff to be collected, utilized, and stored in order to increase groundwater levels. Currently, certain areas of the city are experiencing water shortages during the warm summer months, and the level of groundwater has decreased by a sizeable level.

In order to shed some light on the crucial role that data plays in the preparation of hydrological and hydrodynamic models, we must keep in mind that obtaining data in developing countries is not an easy task. There are difficulties and challenges to overcome at each stage. Even if the goal is to develop a simple flood model, we face obstacles and challenges. During the course of this work, we tested a number of different approaches to prepare the hydrodynamic model. In the end, we were successful in employing the BR technique to construct a model with cell size (10 * 10). In point of fact, we were able to draw the conclusion, on the basis of our observations, that the BR technique is adequate for modelling flash floods in regions where there is a scarcity of data.

## Data Availability

The datasets generated during and/or analysed during the current study are available from the corresponding author on reasonable request.
